# Physician Wellness Measures and Clinical Performance on a Critically Ill Simulated Patient: Does a Lack of Well-Being Impact Patient Care?

**DOI:** 10.7759/cureus.16369

**Published:** 2021-07-13

**Authors:** Cynthia Peng, Linn Lung, Madeline Grade, Nelson Wong, Rebecca Smith-Coggins

**Affiliations:** 1 Emergency Medicine, Stanford School of Medicine, Palo Alto, USA; 2 Emergency Medicine, Kaiser San Rafael Hospital, San Rafael, USA; 3 Emergency Department, University of California San Francisco, San Francisco, USA; 4 Emergency Medicine, VA Palo Alto Health Care System, Palo Alto, USA; 5 Simulation, SimLEARN, Palo Alto, USA

**Keywords:** medical education, critical care simulation, resident physician, clinical performance, emergency medicine

## Abstract

Background/Objective:

Burnout is common among resident physicians, which has the potential to translate into diagnostic and management errors. Our study investigates the relationship between sleepiness, depression, anxiety, burnout, and lack of professional fulfillment with clinical performance during a critically ill patient simulation.

Methods/Approach:

Emergency medicine residents were recruited to participate in a high-fidelity simulation case of a critically ill patient. A survey with validated wellbeing measures (National Institutes of Health Patient-Reported Outcomes Measurement Information System (NIH PROMIS), Linzer burnout measure, and professional fulfillment index) was administered prior to the simulation. Each encounter was video-recorded and analyzed by two blinded raters based on a binary critical-actions checklist. Time-to-intubation, management errors, and misdiagnosis rates were assessed.

Results:

Twenty residents participated, with most subjects endorsing sleepiness (70%) and less than half reporting depression (40%) and anxiety (45%). Burnout was identified to be in 50% of participants by the Linzer measure and 85% by the professional fulfillment index. No significant difference was found between mean performance scores in sleepy, depressed, and anxious cohorts in comparison to groups without those symptoms. Similarly, burnout and professional fulfillment did not yield any significant difference, nor did comparisons with time to intubation, management errors, and frequency of misdiagnosis.

Conclusion:

Resident burnout, depression, anxiety, sleepiness, and lack of professional fulfillment did not appear to have a measurable impact on clinical performance in managing a critically ill patient. There is no evidence from this study that the lack of resident physician well-being adversely impacts patient care by increasing errors in management or misdiagnoses during this high-fidelity simulation.

## Introduction

Burnout has been defined by Dr. Christina Maslach as having three key components: 1) a feeling of overwhelming exhaustion, 2) cynicism and detachment from the job, 3) and a sense of ineffectiveness and lack of accomplishment [1]. These symptoms often begin manifesting early in medical learners during medical school with increasing depersonalization and emotional exhaustion [2,3]. Burnout, as well, is cited as a commonplace syndrome among the United States (US) physicians, with an alarming prevalence of 40-50% in physicians at all career stages [4]. Threats to well-being include factors such as lack of sleep and the co-existence of depression and anxiety [5].

The complex interplay of these entities in trainees may have an impact on clinical performance. In Fahrenkopf et al., significantly more medical errors were made by residents who met the criteria for depression but not burnout [2]. Similarly, in simulated medical scenarios, lower cumulative performance scores on high-fidelity simulation scenarios have been associated with emergency medical trainees exhibiting signs of burnout [6].

The margin for medical errors in high-stakes medical situations, such as that of a critically ill patient, is low. Misdiagnosis, management mistakes, and delay in action can lead to devastating consequences. There has been limited investigation into the relationship between quality of care delivered and metrics of wellness as measured by the physicians in charge of the resuscitation.

Our study attempts to investigate the correlation between wellness deficits reported by emergency medicine residents in sleepiness, depression, anxiety, burnout, and lack of professional fulfillment and their subsequent clinical performance on a simulated critically ill patient encounter.

## Materials and methods

This was a cross-sectional, single-center prospective study in a Level 1 Trauma Center. We recruited a convenience sample of emergency medicine residents, post-graduate year (PGY) one through four, to participate from Jan 2018-March 2019. The study was approved by the Stanford Institutional Review Board (IRB).

All subjects completed a baseline survey, which included wellness assessment instruments as well as basic demographic information. Items included the short form version for National Institutes of Health Patient-Reported Outcomes Measurement Information System (NIH PROMIS) measures of depression, sleep-related impairment, and anxiety [7]. Burnout was measured with a psychometrically validated single-item question from the Maslach Burnout Inventory and the Professional Fulfillment Index (PFI) scale [8,9].

Each subject participated in a high-fidelity simulation case involving a critically ill patient: a patient presenting to the Emergency Department (ED) from a house fire with carbon monoxide and cyanide poisoning, which required rapid sequence intubation and initiation of other therapies. A binary critical-actions checklist was created based on the expert consensus of three practicing emergency physicians. An audiovisual recording was collected of each simulation session. The simulation session was run by two simulation-trained emergency medicine faculty.

Two independent raters with clinical medical backgrounds and simulation training were blinded to the identity and experience level of the participants. They rated each trainee’s simulation performance based on an itemized checklist (maximum score of 21). Discrepant performance items were resolved by a third reviewer. Additional data measured included time-to-intubation, management error, and misdiagnosis rates. Management error was defined as missing greater than or equal to one critical intervention and/or treatment. Misdiagnosis was broadly defined as missing or having the incorrect final diagnosis.

Statistical analysis on survey results and simulation performance was performed using SPSS version 25 (IBM Inc., Armonk, USA). Descriptive statistics were analyzed, and the normality of the dependent variables was assessed using the Shapiro-Wilk test. Simulation scores and time to intubation were compared to the NIH PROMIS measures using a parametric independent t-test with a significance level of 0.05. For the other well-being measures that did not have normally distributed data, the Kruskal-Wallis and Wilcoxon rank-sum test was utilized.

## Results

Twenty residents were recruited and participated in the study (Table 1). Of the participants, 68% were male, and 32% were female. 45% representing the PGY2 class and 55% PGY3 class. 63% of the participants were married or in domestic partnerships, and 75% reported Caucasian ethnicity.

**Table 1:** Descriptive characteristics of participants *incomplete entries were excluded from designated categories

Characteristic	Frequency
Age*	
< or =30 years old	10 (56%)
>30 years old	8 (44%)
Training Year	
PGY2	9 (45%)
PGY3	11 (55%)
Gender*	
Female	6 (32%)
Male	13 (68%)
Ethnicity	
Caucasian	15 (75%)
Other	5 (25%)
Family status*	
Single	7 (37%)
Married or domestic partnership	12 (63%)
Children	4 (21%)

The majority of participants (70%, n=14) endorsed sleepiness, and 40% screened positive for depression (n=8) and 45% for anxiety (n=9) (Table 2). There were no differences in burnout measures between male and female groups. Burnout was identified in 50% of participants (n=10).

**Table 2:** Wellness metric measures and participant responses

Characteristic	Frequency
Sleepiness	
Y	14 (70%)
N	6 (30%)
Depression	
Y	8 (40%)
N	12 (60%)
Anxiety	
Y	9 (45%)
N	11 (55%)
Burnout	
Y	10 (50%)
N	10 (50%)
Professional Fulfillment	
Y	12 (60%)
N	8 (40%)
Professional Fulfillment Burnout	
Y	17 (85%)
N	3 (15%)

No significant difference was found in mean simulation performance scores between the sleepy versus not sleepy cohorts (15 vs. 15.5). This finding was congruent for depression and anxiety measures as well (Table 3). Similarly, burnout versus no burnout groups and professionally fulfilled versus not fulfilled groups did not yield any significant difference in simulation performance scores. There was also no significant difference in time to intubation, management errors, and frequency of misdiagnosis between all wellness measure groups.

**Table 3:** Composite simulation performance measures versus wellness measures

	Simulation Score	Time to Intubation (seconds)	>1 Management error	>1 Misdiagnosis
Sleepiness				
Y	15 ± 2.5	294.9 ± 169.9	85.7%	55.6%
N	15.5 ± 1.1	274.8 ± 57.3	66.7%	33.3%
Depression				
Y	15 ± 2.9	250.9 ± 108.1	75.0%	25.0%
N	15.3 ± 1.5	314.3 ± 163.0	83.3%	41.7%
Anxiety				
Y	15.6 ± 2.4	288.1 ± 91.3	77.8%	22.2%
N	14.8 ± 1.9	289.5 ± 180.7	81.8%	45.5%
Burnout				
Y	16 ± 1.8	331.3 ± 171.4	80.0%	20.0%
N	14.3 ± 2.2	246.5 ± 101.6	80.0%	50.0%
Professional Fulfillment				
Y	14.8 ± 2.4	263.8 ± 103.5	75.0%	41.7%
N	15.6 ± 1.6	326.5 ± 191.5	87.5%	25.0%
Professional Fulfillment Burnout				
Y	15 ± 2.2	289.7 ± 156.5	82.4%	35.3%
N	16 ± 1	284.3 ± 25.4	66.7%	33.3%

Subanalysis revealed no difference in PGY2 and PGY3 simulation scores (15.5 vs. 14.8), time to intubation (241 seconds vs. 327 seconds), management error (77.8% vs. 81.8%), or frequency of misdiagnosis (33.3% vs. 36.4%). Below-average performers (under a score of 15) did not display more signs of sleepiness, depression, anxiety, or burnout compared to peers that performed better.

## Discussion

In this simulation case of a critically ill patient, emergency medicine resident performance was not significantly impacted by their self-reported sleepiness, depression, anxiety, burnout, or professional fulfillment. Importantly, there were no significant delays in intubation, increase in management errors or additional critical misdiagnoses. With simulation performance as a proxy for clinical performance, our findings suggest that providers may be able to perform adequately when caring for individual critically ill patients even in the context of scoring lower measures on various wellness metrics.

Overall, the rates of depression, anxiety, and burnout in this study cohort were fairly similar to meta-analysis prevalence data among resident physicians. Prevalence of all levels of depressive symptoms among residents broadly ranges from 20.9% to 43.2% [10].

The cognitive load from taking care of critically ill patients may be higher than for lower acuity patient care. However, in the face of a rapidly decompensating patient, it may require the full engagement of the physician to stabilize the crisis. Our study suggests that the lack of wellness may not be evident in this scenario and does not translate to negligent care. This is concordant with prior limited work in this area [6]. Further studies will be needed to investigate if this is also similar in settings of routine patient care or in the care of multiple critical patients.

Our study highlights the adaptive nature of our medical learners. This perhaps showcases the self-selection into medicine of individuals with underlying resilient traits. The adversities and stressors encountered during years of medical training may promote the development of resilience [11]. This may allow trainees to retain high performance even when experiencing symptoms of depression, anxiety, sleepiness, and burnout. Interestingly, learners who had below-average performances did not report worsened wellness across our metrics. This further supports our findings that lack of well-being is not necessarily related to performance.

## Conclusions

In conclusion, resident burnout, depression, anxiety, sleepiness, and lack of professional fulfillment did not appear to have a measurable effect on overall clinical performance on this high-fidelity simulation of a critically ill patient. 

Our study was not without limitations. We had a strictly defined cohort of emergency medicine residents from a single center. In addition, the sensitivity of the measures of performance within our specific simulation was not previously validated in the literature and is difficult to generalize to the care of all critically ill patients. In addition, certain wellness metrics, such as the NIH PROMIS measures, were developed based on the general population and not for our specific cohort. Our study invites future studies to further delineate the relationship between wellness and care of critically ill patients with a larger sample of learners or more complex simulations. 

Further discrimination between determinants of wellness and their effect on clinical performance outside of the simulated setting is warranted. In addition, the direct implications of this on patient care outcomes is a fertile area for future investigations.
